# Association of Sleep Disordered Breathing with Mono-Symptomatic Nocturnal Enuresis: A Study among School Children of Central India

**DOI:** 10.1371/journal.pone.0155808

**Published:** 2016-05-18

**Authors:** Bharat Choudhary, Rajesh Patil, Girish Chandra Bhatt, Abhijit P. Pakhare, Abhishek Goyal, Aswin P, Bhavna Dhingra, K. C. Tamaria

**Affiliations:** 1 Department of Pediatrics, All India Institute of Medical Sciences (AIIMS), Bhopal, India; 2 Department of Community & Family Medicine, All India Institute of Medical Sciences (AIIMS), Bhopal, India; 3 Department of Pulmonology and Sleep Medicine, All India Institute of Medical Sciences (AIIMS), Bhopal, India; Postgraduate Institute of Medical Education and Research, INDIA

## Abstract

**Objective:**

To study the prevalence of primary monosymptopomatic nocturnal enuresis (PMNE) in children aged 5–10 year and to find its association with sleep disordered breathing (SDB) by using a 22 item pediatric sleep related breathing disorder (SRBD) scale.

**Methods:**

This was a school based cross sectional epidemiological study from July 2015 to November 2015. A questionnaire seeking information on socio-demographic variables, nocturnal enuresis (NE) frequency, school performance and a validated 22 item pediatric sleep related breathing disorder scale (SRBDs) was distributed to 1820 pupils in three primary schools.

**Results:**

A total of 1528(83.95%) questionnaires were retrieved. Out of 1528 forms, 182(11.9%) forms were incomplete for requested information and hence 1346 (73.9%) questionnaires were finally analyzed. The prevalence of NE was found to be 12.7% (95% CI; 11–14.6), whereas prevalence of primary nocturnal enuresis (PMNE) was 8.2% (95% CI; 7.1–10.1). SRBD scale score >0.33 (adjusted OR: 2.87; 95%CI: 1.67–4.92), paternal history of enuresis in childhood (adjusted OR:4.96; 95% CI: 2.36–10.45), and inappropriate toilet training (adjusted OR: 1.64; 95% CI: 1.01–2.66) were independently associated with PMNE.

**Conclusion:**

Sleep disordered breathing, inappropriate toilet training and a history of childhood NE in father were found to be significant risk factors for PMNE in the present study. Thus, these findings suggest that it is imperative to rule out SDB in PMNE patients as they may require different therapeutic interventions.

## Introduction

Enuresis is one of the prevalent elimination disorders in childhood. American psychiatric association (DSM-V) defines enuresis as bedwetting in children ≥ 5 years after exclusion of organic causes[1]. Further, it must be clinically significant as manifested by either frequency of at least twice a week for at least 3 consecutive months or the presence of clinically significant distress or impairment in social, academic (occupational) or other important areas of functioning. The chronological age or the developmental age must be equivalent to 5 years and the behavior should not be attributable to the physiological effects of a substance or any medical condition. Monosymptomatic nocturnal enuresis (MNE) is defined as enuresis in night only and without any lower urinary tract symptoms like urgency, dribbling, increased frequency or hesitancy[2]. Various studies across the globe have shown the prevalence of enuresis ranging from 4–15%. However, the prevalence of MNE ranged from 9–12% in these studies[3][4][5][6][7]. Nocturnal polyuria, sleep disturbances, genetic factors, impaired psychological maturation. and bladder dysfunction are major underlying factors responsible for MNE.

Obstructive sleep apnea (OSA) in adults is associated with increased enuresis prevalence. Similarly, prevalence of sleep related problems (including OSA and frequent awakenings) among children diagnosed with MNE ranged from 25–30%[8][9]. These results are based on a few hospital based studies and larger community based studies are lacking[8][10]. Thus, in this study we tried to explore the association between nocturnal enuresis and sleep disordered breathing (SDB) in school going children of Central India, aged 5–10 years by using a validated pediatric sleep related breathing disorder(SRBD) questionnaire[11].

## Methodology

### Study type and set up

A school based cross sectional epidemiological study, was done at three primary schools of Bhopal, Madhya Pradesh from July 2015-November 2015. Children aged between 5 to 10 years in three schools were enrolled. An English medium and two Hindi medium schools were purposively selected. The study was approved by the Institutional human Ethics Committee of All India institute of Medical Sciences (AIIMS), Bhopal (Ref letter no.IHEC-LOP/2015/IM007). We obtained written informed consent from the parents of the children who participated in the study. A note regarding the identification of the symptoms of polysymptomatic nocturnal enuresis and secondary enuresis was given at the end of each questionnaire.

### Sampling procedure and Data Collection

To estimate the prevalence of primary monosymptopomatic nocturnal enuresis (PMNE) of 12.5% (as reported in previous studies) with 95% CI (confidence interval) of 10–15% and design effect of 2, calculated sample size was 1400. Since, questionnaire was sent to parents through students, we anticipated higher non response rate of 30%. Thus final sample size was 1820.

Study objectives and detailed procedure were explained to the heads of the selected schools and class teachers prior to initiation of the study. Students were given questionnaire during their class and were instructed to get it filled by their parents, if they were willing for participation. Along with the questionnaire an informed consent and participant information sheet were also provided. Questionnaire had sought information regarding socio-demographic variables, nocturnal enuresis frequency, school success, anthropometry (S1 File) and a 22 item pediatric sleep related breathing disorder scale (SRBDs) (S2 File)[11]). Retrieval of the questionnaire from the students was done within 5–7 days by the investigators.

### Definitions

Co- sleeping meant sleeping at night with at least one of the parents.

Toilet training was defined as either appropriate or inappropriate. Training given by reward method was considered appropriate, while toilet training given by punishment or fear was considered inappropriate. Children with history suggestive of renal disease, neurological disease, on medications known to induce diuresis, incomplete forms, age less than 5 years were excluded from the study.

### Translation of SRBD scale

SRBD scale was adapted from university of Michigan after permission. This scale has been validated with Polysomnography for sleep disordered breathing. Hindi translation was done after due permission. For Hindi translation, English version of questionnaire was given to five bilingual pediatricians and they were asked to translate in Hindi. After this, a consensus meeting was held and an initial Hindi version was prepared. The Hindi version was further back translated into English by another bilingual pediatrician. Any discrepancy in the two versions was then resolved by consensus. The questionnaire was then given to parents of 30 children attending Pediatric outpatient department (OPD). They were asked to fill in the questionnaire and report any ambiguity or confusion in questions. Subsequently, a final Hindi version of the scale was contrived after getting due feedback from parents.

### Interpretation of SRBD scale

SRBD scale contains 22 symptom items about snoring frequency, loud snoring, difficulty breathing during sleeping, observed apneas, daytime sleepiness, inattentive or hyperactive behavior etc. Each of these items was shown to correlate with OSA in children confirmed by Polysomnography[12]. There are three option to answer each question in the scale—yes = 1, no = 0, or don’t know = missing. The number of symptom-items endorsed positively (“yes”) is divided by the number of items answered positively or negatively; the denominator therefore excludes items with missing responses and items answered as don’t know. The result is a proportion that ranges from 0.0 to 1.0. Scores > 0.33 are considered positive and suggestive of high risk for a pediatric sleep-related breathing disorder.

### Statistical analyses

Statistical analysis was done using Epi-Info -7 software. Prevalence is reported as a proportion with 95% confidence interval. Comparison of different socio-demographic and other variables among children with and without nocturnal enuresis was done by chi-square test and unpaired t-test/Mann Whitney test appropriately. Statistically significant and biologically important variables were then entered in logistic regression model to identify independent predictors of nocturnal enuresis. Stepwise forward conditional model was used for identifying independent predictors. Hosmer-Lemeshow Goodness of Fit test was used for testing fit of model. A p-value less than 0.05 was considered as statistically significant. Univariate logistic regression analysis was performed to test association of various risk factors with nocturnal enuresis. Then statistically significant factors (Table 1) and certain known predictors such as co-sleeping, overweight and sleeping time after 10 pm were also included in multivariate logistic regression model. The results of the multivariable analysis are reported as adjusted odds ratios (adj OR) with 95% CI.

**Table 1:** Distribution of socio-demographic and clinical variables.

Variable		PMNE		Normal		Total	p-value
		n	%	n	%		
Gender	Male	59	8.1	666	91.9	725	0.486
	Female	41	9.3	399	90.7	440	
Age Group	< = 6.00	28	11.7	211	88.3	239	
	6.01–7.00	20	9.3	194	90.7	214	
	7.01–8.00	23	8.3	255	91.7	278	0.252
	8.01–9.00	16	6.4	233	93.6	249	
	9.01+	9	6.6	128	93.4	137	
Handedness	Left	17	7.4	213	92.6	230	0.447
	Right	90	9.0	915	91.0	1005	
Education of Father	Illiterate	3	9.4	29	90.6	32	0.915
	Literate	111	8.8	1146	91.2	1257	
Education of Mother	Illiterate	8	12.5	56	87.5	64	0.291
	Literate	106	8.7	1119	91.3	1225	
Co-Sleeping	No	2	10.0	18	90.0	20	0.854
	Yes	112	8.8	1157	91.2	1269	
ToiletTrainingType	Appropriate	87	8.0	1000	92.0	1087	0.014
	Inappropriate	27	13.4	175	86.6	202	
Exclusive Breast Feeding	Yes	78	8.7	817	91.3	895	0.806
	No	36	9.1	358	90.9	394	
SiblingBirth	Yes	14	14.4	83	85.6	97	0.055
	No	95	8.6	1011	91.4	1106	
Weight for Age (Z-score)	< = -2.00	12	6.8	164	93.2	176	
	-1.99–2.00	62	8.1	706	91.9	768	0.637
	2.01+	4	11.4	31	88.6	35	
SRBD_33	< = 33.0%	89	7.6	1085	92.4	1174	<0.001
	33.1%+	25	21.7	90	78.3	115	
H/o Enuresis in Father	Yes	14	31.8	30	68.2	44	< .000
	No	100	8.0	1145	92.0	1245	
H/o Enuresis in Mother	Yes	4	16.7	20	83.3	24	0.173
	No	110	8.7	1155	91.3	1265	
Sleep Duration	< = 9.00	32	8.2	360	91.8	392	0.578
	9.01+	49	9.2	483	90.8	532	
Sleeping Time	< 22.00	31	7.2	399	92.8	430	0.113
	22.00+	80	9.9	727	90.1	807	

## Results

Out of the 1820 questionnaires distributed, 1528 were retrieved, resulting in response rate of 83.95%. Out of 1528 forms, 182(11.9%) forms were incomplete for requested information and hence 1346 (73.9%) questionnaires were finally analyzed.

### Demographic characterization

Table 1 depicts association of various demographic and clinical factors with nocturnal enuresis. 62% of children were boys. Mother and father were informants for 53.5% and 39% of the children respectively.

### Prevalence of Enuresis

The prevalence of NE was found to be 12.7% (95% CI; 11–14.6), whereas prevalence of PMNE was 8.2% (95% CI; 7.1–10.1). PMNE was more common among children who were 6 years old and there was declining trend in prevalence of PMNE with age. Primary non-monosymptomatic or polysymptomatic enuresis was found in 9[0.5% (95% CI; 0.35–1.6)] children though secondary enuresis was found in 48 [3.6% (95%CI; 2.7–4.7)] children.

### Risk factors for PMNE

SRBD score was positive in 8.9% (95%CI; 7.5–10.6) of the children, while 21.9% (95% CI; 15.3–30.4) of children with PMNE had positive SRBD score. On univariate analysis a SRBD score >0.33[OR 3.38, (95% CI -2.06–5.54)], history of enuresis in father [OR 5.34, 95% CI (2.74–10.4) and inappropriate toilet training [OR 1.77, (95% CI -1.11–2.81)] were statistically significant risk factors for PMNE. Recent sibling birth reached a borderline level of statistical significance [OR 1.79(95% CI; 0.98–3.28)](Table 1). On multivariate analysis by forward conditional method, only SRBD score >0.33 (adjusted OR: 2.87; 95%CI: 1.67–4.92), history of enuresis in father (adjusted OR:4.96; 95% CI: 2.36–10.45) and inappropriate toilet training (adjusted OR: 1.64; 95% CI:1.01–2.66 -) were statistically significant (Table 2).

**Table 2:** Univariate and multivariate logistic regression analysis for PMNE.

Variable	Univariate Analysis				Multivariate Logistic Regression			
	OR	95% C.I for OR		p-value	OR	95% C.I adj.OR		p-value
		Lower	Upper			Lower	Upper	
Sleeping Time> 22:00	1.41	0.91	2.18	0.113	1.32	0.84	2.07	0.150
SRBD Score > 33	3.38	2.06	5.54	<0.001	2.87	1.67	4.92	<0.001
Inappropriate Toilet Training	1.77	1.11	2.81	0.013	1.64	1.01	2.66	0.048
Recent Sibling Birth in Family	1.79	0.98	3.28	0.054	1.64	0.87	3.07	0.122
H/ o Enuresis in Father	5.34	2.74	10.4	<0.001	4.96	2.36	10.45	<0.001
H/o Enuresis in Mother	2.1	0.70	6.25	0.173	1.071	0.276	4.15	0.921

PMNE had no effect on—school performance such as overall school success, grades in various subjects (Mathematics, English language, Hindi language, Drawing, Physical education), day lethargy and general behavior of the child (Table 3).

**Table 3:** Comparison of School Performance of Children with and without Enuresis.

Variable		PMNE		Normal		Total	P-value
		N	%	N	%		
School success (Grades)	A	44	38.9	481	43.3	525	
	B	45	39.8	427	38.5	472	0.371
	C	22	19.5	162	14.6	184	
	D	2	1.8	40	3.6	42	
Math	Good	87	76.3	880	74.9	967	0.738
	Poor	27	23.7	295	25.1	322	
Science	Good	69	60.5	724	61.6	793	0.819
	Poor	45	39.5	451	38.4	496	
Hindi	Good	85	74.6	882	75.1	967	0.906
	Poor	29	25.4	293	24.9	322	
English	Good	89	78.1	882	75.1	971	0.477
	Poor	25	21.9	293	24.9	318	
	Drawing	78	68.4	796	67.7	874	0.883
		36	31.6	379	32.3	415	
Physical Education	Good	76	66.7	768	65.4	844	0.780
	Poor	38	33.3	407	34.6	445	
Day lethargies	Yes	12	11.2	90	8.1	102	0.265
	No	95	88.8	1022	91.9	1117	
Child Behavior	Extroverted	75	67.6	805	71.4	880	0.220
	Introverted	4	3.6	37	3.3	41	
	Sensitive can get hurt easily	10	9.0	117	10.4	127	
	Shy	8	7.2	86	7.6	94	
	Aggressive	13	11.7	63	5.6	76	
	Hurts others and doesn't feel sorry	1	.9	19	1.7	20	

## Discussion

Studies around the world have shown that nocturnal enuresis has significant prevalence among children. This study revealed prevalence of NE and PMNE to be 12.7% and 8.2% respectively. Previous studies have shown prevalence of PMNE ranging from 3.8% to 18.9%[13]. This wide variation in the prevalence may be attributed to cultural, racial, environmental, varying case definition and age group criteria among different studies.

In most of the studies male children had higher prevalence of enuresis compared to females, similarly in our study male: female ratio was 1.4:1.Age, obesity, co -sleeping, recent sibling birth, poor scholastic performance, inappropriate toilet training were found to be significantly associated with PMNE in various studies[7][14][15][16]. In this study, children with inappropriate toilet training, family history of enuresis in father and SRBD score >0.33 were found to have higher odds for developing PMNE. Children who were given toilet training by punishment or threat have 1.6 times higher odds of associated PMNE as compared to those who received toilet training by reward method. Previous studies have also shown inappropriate toilet training method as an important risk factor for nocturnal enuresis[17][18][19].Some of the studies reported recent sibling birth as a risk factor for enuresis but no significant association was observed in our study[19]^.^

Family history of enuresis has been implicated as a risk factor for PMNE in most of the studies[2][19]. Similarly, in the present study there was a fivefold higher odds of having PMNE in a child whose father had history of childhood NE. This has been attributed to the well understood genetic background of NE in various models[20].

In addition to above risk factors, SRBD score >0.33 was found to be strongly associated with PMNE. In the present study there is a threefold association between PMNE in the children and positive SRBD. The 22 item SRBD scale has been validated for use in research studies for the assessment of obstructive sleep apnea(OSA) and the instrument has shown good internal consistency and test retest reliability[12].

Previous small hospital based studies have also shown association of OSA and nocturnal enuresis[3][21][22]. A study by Alexopoulos, et al. found that NE was associated significantly with presence of moderate-to-severe OSA after adjustment for tonsillar hypertrophy, obesity, gender, and age (adjusted odds ratio = 1.92 (1.08–3.43); P = 0.03). Another study using overnight polysomnography in 43 children with MNE against 30 controls reported, higher prevalence of OSA in children with refractory NE[23]. Waleed, et al. in a hospital based study, diagnosed sleep disordered breathing in 30.4% of enuretic children with a downgrade trend of enuresis and SRBD with increasing age[8]. A systematic review of 14 studies further revealed that sleep disordered breathing is associated with nocturnal enuresis and there was a significant improvement in enuresis in the children who underwent adenotonsillectomy[9]. Kovacevic, et al. prospectively evaluated 46 children with polysomnography confirmed OSA and MNE requiring adenotonsillectomy to release upper airway obstruction and concluded that 43.5% of children became dry after adenotonsillectomy[22]. Based on these findings, it is suggested to screen children with MNE for the presence of OSA by appropriate scales as they may require different therapeutic interventions[24].

There are conflicting patho-physiological pathways that have been attributed to increased frequency of enuresis in OSA patients. Some studies have implicated a possible role of Brain-type natriuretic peptide (BNP) which is released from cardiac myocytes after cardiac wall distension. OSA could lead to increased urinary output and natriuresis by promoting the release of BNP[8][25]. On the other hand, some of the studies did not find any association between natriuretic peptide and OSA in the adult patients[26][27].

The strengths of our study are: 1) our study is one of the few community based studies exploring the association between OSA and MNE in the children aged 5–10 years. Our study further confirmed a high prevalence of OSA in patients with MNE (21.9%) as compared to the general pediatric population (8.9%). Moreover, previous studies have also shown a significant higher rates of OSA in patients with refractory nocturnal enuresis[23]. Thus, the study findings suggests that it is imperative to screen the children with MNE for the presence of symptoms of OSA by appropriate scales, as the patients of MNE with concomitant OSA may require different therapeutic interventions.

The study has certain limitations: As we have used SRBD questionnaire for the identifications of the OSA in this study, the overestimation of the OSA in this study is likely. Further, a formal validation of the translation of the SRBD scale was not performed and we have not confirmed these findings with the gold standard polysomnography in our patients. Nevertheless, in the settings where polysomnography is neither available nor feasible, the SRBD subscale of the pediatric sleep questionnaire has been shown to be both reliable and valid in identifying SDB in children in clinical research[28].Also, despite cleaning of data and discarding highly incomplete forms, there were some variables for which informants have not given response (Table 1) but these forms were almost complete for the required information, and therefore were included in analysis. Another potential limitation of the study may be the overestimation of the prevalence of NE due to inclusion of the children with undiagnosed urinary disease with no other symptom apart from NE.

## Conclusion

Sleep disordered breathing, inappropriate toilet training and a history of childhood NE in father were found to be significant risk factors for PMNE in the present study. Thus, these findings suggest that it is imperative to rule out SDB in PMNE patients as they may require different therapeutic interventions.

## Ethical consideration

Parents of children with symptoms of polysymtomatic or secondary enuresis were advised to visit outpatient department (OPD) for detailed evaluation. Moreover, at the end of the study a counseling session was done by the Pediatricians involved in the study with the parents of the children with positive SRBD scale and enuresis. They were instructed to visit OPD for detailed evaluation.

## Supporting Information

S1 File Nocturnal enuresis questionnaire. (DOC)

S2 File Sleep Related Breathing Diosrder (SRBD) questionnaire. (DOC)

## Abbreviations

NE: Nocturnal enuresis
SDB: Sleep disordered breathing
PMNE: Primary monosymptomatic nocturnal enuresis
SRBD: Sleep related breathing disorder
MNE: Monosymptomatic nocturnal enuresis
OSA: Obstructive sleep apnea
