# Supplementary material for: Association of Sleep Disordered Breathing with Mono-Symptomatic Nocturnal Enuresis: A Study among School Children of Central India
Source: PLoS One. 2016 May 18;11(5):e0155808. doi: 10.1371/journal.pone.0155808 (PMC4871538; doi:10.1371/journal.pone.0155808)
Supplement: S1 File — (DOC) [file pone.0155808.s001.doc]

**Interview of parents/caregivers to assess prevalence of NE**

Name-

Weight (kg)-

Height (cms)-

BMI-Your doctor will calculate this.

Encircle appropriate option.

1. Informant
2. Mother B. Father C. Others
3. Does your child have been completely dry by night (Not urinating in bed) for at least 6 months after 5 years of age.
4. Yes
5. No
6. Does your child passes urine in bed during night
7. Yes
8. No
9. If yes how frequently
10. Less than 2 times in a week
11. 2 or more times a week
12. For how many months this is persisting
    A. less than 3 months
    B. 3 months or more
13. Circle age of your child
    A.3 B.4 C.5 D.6 E.7 F.8 G.9 H.10
14. Sex
    A. Male B. Female
15. At what age was your child toilet trained for wetting?
    A. 18 months B.2 yrs C. 2 1/2 yrs D. 3 yrs E. 3 1/2 yrs F. 4 yrs.
16. Does your child has following problems(encircle the problem)
    1. Passes urine more than 8 times a day(frequency)
    2. Passes urine less than 4 times a day
    3. Bedwetting during daytime also
    4. Cannot hold urine(Urgency)
    5. Burning micturition
    6. Passes urine on cloths during daytime also.
    7. Undergarments remains wet most of the time
    8. Remains constipated most of the time

Any other please specify…………………….

1. Does your child developed bedwetting(> 5 years of age) after at least six months of bladder control
   1. Yes
   2. No

1. The Child lives in
   A. Hostel B. House (Day scholar)

1. Birth order
   A. 1^st^ B. 2^nd^ C. 3^rd^ D. 4^th^ E. greater than 4
2. History of recent sibling born in family
3. Yes B. No
4. How toilet training was achieved in this child
5. Reward
6. Punishment
7. Threat
8. "Other, Please specify"
9. For how many months the child was given only breast milk and not even water during infancy
10. 2 months
11. 3-4 months
12. 5-6 months
13. For how many years breastfeeding was continued in this child
14. <6 months
15. 6-11months
16. 12-17 months
17. 18-24 months
18. >24 months
19. Fathers education
    A. Illiterate
    B. Read and write/ Primary Education
    C. Secondary Education
    D. Higher secondary Education
    E. Graduation/ Post- graduation
20. Mothers education
    A. Illiterate
    B. Read and write/ Primary Education
    C. Secondary Education
    D. Higher secondary Education
    E. Graduation/ Post- graduation
21. Are both parents working
    A. Yes B. No
22. History of Enuresis in father
    A. Yes B. No C. Don’t know
23. History of enuresis in mother
    A. Yes B. No C. Don’t know
24. Type of family
    A. Nuclear B. Combined
25. History of enuresis in father
    A. Yes B. No
26. Total income of family (in rupees)
    A. 1520 or below
    B. 1521- 4555
    C. 4556- 7593
    D. 7594-11361
    E. 11362-15187
    F. 15188- 30374
    G. 30375 or above

**Questions regarding behavior /sleep pattern of child**

1. Handedness of child
2. Left Handed B. Right Handed
3. Child's general behavioral attributes
4. Extroverted ( comfortable with social interactions)
5. Introverted ( lacks interest or is uncomfortable with social interactions)
6. Sensitive, can get hurt easily
7. Shy
8. Aggressive
9. Hurts others and do not feel sorry about it.
10. Parent's behavior towards the child
11. Normal
12. Protective
13. Harsh
14. Setting free
15. Indifferent
16. Grade of your child in following subjects in half yearly/final exams

Maths.........(A / B / C /D ) Hindi………......... (A / B/ C /D) Science……...(A / B / C /D ) English……….... .(A / B/ C /D)

Drawing…….. (A / B / C /D ) Physical Education……...... (A / B / C /D)

1. School success
2. Very successful (A+, A / 80%-100%)
3. Successful (B+, B / 68%-79%)
4. Intermediate (C+, C/ 55%-67%)
5. Low (D/ 50 %-54%)
6. Very low (below D/ less than 50%

**"Does your child have sleep problem, if yes please indicate"**

1. Difficulty with falling asleep
2. Present B. Absent
3. Sleep talking
4. Present B. Absent
5. Sleep bruxism
6. Present B. Absent
7. Sleep walking
    A. Present B. Absent
8. Deep sleep
9. Present B. Absent
10. Waking up in night
11. Present B. Absent
12. Day lethargies
    A. Present B. Absent
13. Any family history of seizures?
    A. Present B. Absent
14. Day time ** sleeping?
    A. Present B. Absent
15. Was there a delay in achieving developmental milestones?
    A. Yes B. No
16. Circle if there is a family history of any of the following diseases.
    A. Diabetes B. Renal disorders C. Nocturnal Enuresis
17. Does your child sleeps alone or has a habit of co-sleeping with siblings/parents
    1. Sleeps alone B. Co-sleeps
18. Till which age Diaper was used in your child (Write down exact age in months,e.g 1year 4 months etc).
19. Is your child taking any medications?
    1. Yes
    2. No
20. If yes, indicate which medications he is on………………………..

***Note***: ** Any participant with daytime symptoms like urgency, incontinence, or day time sleeping is recommended to visit pediatrics department of AIIMS Bhopal OPD or a nearby physician.
