# Supplementary material for: Association of Sleep Disordered Breathing with Mono-Symptomatic Nocturnal Enuresis: A Study among School Children of Central India
Source: PLoS One. 2016 May 18;11(5):e0155808. doi: 10.1371/journal.pone.0155808 (PMC4871538; doi:10.1371/journal.pone.0155808)
Supplement: S2 File — (DOC) [file pone.0155808.s002.doc]

# **Child’s Name: ______________________________ Subject no. #: ___________**

**Person completing form: _____________________ Date: ____/____/____**

**Please answer these questions regarding the behavior of your child during sleep and wakefulness. The questions apply to how your child acts in general during the past month, not necessarily during the past few days since these may not have been typical if your child has not been well. You should circle the correct response or *print* your answers neatly in the space provided. A “Y” means “yes,” “N” means “no,” and “DK” means “don’t know.”**

1. WHILE SLEEPING, DOES YOUR CHILD:

## Snore more than half the time?…………………………………………. ………..Y N DK

Always snore? ………………………………………………………………..Y N DK

Snore loudly? …………………………………………………………………Y N DK

Have “heavy” or loud breathing? …………………………………….…………..Y N DK

Have trouble breathing, or struggle to breathe? …………………….……………Y N DK

# HAve you ever seen your child stop breathing during

# the night? ……………………………………………………………………….Y N DK

3. DOES YOUR CHILD:

Tend to breathe through the mouth during the day?…………………………….Y N DK

Have a dry mouth on waking up in the morning? ……………………………...Y N DK

Occasionally wet the bed? ……………………………………………………...Y N DK

4. DOES YOUR CHILD:

Wake up feeling unrefreshed in the morning? ………………………………….Y N DK

Have a problem with sleepiness during the day? ………………….……….…...Y N DK

1. Has a teacher or other supervisor commented that your

child appears sleepy during the day? …………………………….…Y N DK

1. Is it hard to wake your child up in the morning? …………….…Y N DK

7. Does your child wake up with headaches in the morning?…..Y N DK

1. Did your child stop growing at a normal rate at

any time since birth? …………………………………………………….….Y N DK

9. Is your child overweight? ………………………………………………...Y N DK

10. THIS CHILD **OFTE**

Does not seem to listen when spoken to directly. ……………………………....Y N DK

Has difficulty organizing tasks and activities. …………………….…………...Y N DK

Is easily distracted by extraneous stimuli. ………………………….…………...Y N DK

Fidgets with hands or feet or squirms in seat. ……………………………….....Y N DK

Is “on the go” or often acts as if “driven by a motor”. …………………………Y N DK

Interrupts or intrudes on others (eg., butts into conversations or games). ………Y N DK

**Thank you!**

© Regents of the University of Michigan 2006
